# Development and validation of a practical machine-learning triage algorithm for the detection of patients in need of critical care in the emergency department

**DOI:** 10.1038/s41598-021-03104-2

**Published:** 2021-12-15

**Authors:** Yecheng Liu, Jiandong Gao, Jihai Liu, Joseph Harold Walline, Xiaoying Liu, Ting Zhang, Yunyang Wu, Ji Wu, Huadong Zhu, Weiguo Zhu

**Affiliations:** 1 Emergency Department, State Key Laboratory of Complex Severe and Rare Diseases, Peking Union Medical College Hospital, Chinese Academy of Medical Science and Peking Union Medical College, Beijing, China; 2 Department of Electronic Engineering, Tsinghua University, Beijing, China; 3 Center for Big Data and Clinical Research, Institute for Precision Medicine, Tsinghua University, Beijing, China; 4 Accident and Emergency Medicine Academic Unit, The Chinese University of Hong Kong, Prince of Wales Hospital, Shatin, Hong Kong China; 5 Department of Information Center, State Key Laboratory of Complex Severe and Rare Diseases, Peking Union Medical College Hospital, Chinese Academy of Medical Science and Peking Union Medical College, Beijing, China

**Keywords:** Diseases, Health care, Medical research, Signs and symptoms

## Abstract

Identifying critically ill patients is a key challenge in emergency department (ED) triage. Mis-triage errors are still widespread in triage systems around the world. Here, we present a machine learning system (MLS) to assist ED triage officers better recognize critically ill patients and provide a text-based explanation of the MLS recommendation. To derive the MLS, an existing dataset of 22,272 patient encounters from 2012 to 2019 from our institution’s electronic emergency triage system (EETS) was used for algorithm training and validation. The area under the receiver operating characteristic curve (AUC) was 0.875 ± 0.006 (CI:95%) in retrospective dataset using fivefold cross validation, higher than that of reference model (0.843 ± 0.005 (CI:95%)). In the prospective cohort study, compared to the traditional triage system’s 1.2% mis-triage rate, the mis-triage rate in the MLS-assisted group was 0.9%. This MLS method with a real-time explanation for triage officers was able to lower the mis-triage rate of critically ill ED patients.

## Introduction

Emergency department (ED) overcrowding and resulting delayed medical care are worldwide problems^1^ leading to increased patient mortality^2^. Emergency triage systems can be used to stratify patients’ clinical severity and thus distribute medical resources appropriately. There are various emergency triage systems in current use around the world. The United States widely uses the Emergency Severity Index (ESI), a five-tier triage system^3^. At present, the People’s Republic of China is using a four-tier triage system, the emergency triage scale/standard (ETS), issued by the Chinese National Ministry of Health in 2011^4^. Very much like in the ESI, patients triaged into ETS levels 1 or 2 will likely need critical care and are sent directly to a resuscitation room. Patients triaged into ETS levels 3 or 4 will be considered non-emergent and will have to wait to see a consulting physician. The ETS triage system is generally accurate in most cases and has been widely used in China^5^. However, there are still some critical patients triaged into ETS levels 3 or 4 that need to wait in line with other non-critical patients until they are identified in subsequent medical encounters, sometimes several hours after the initial triage screening. So far there is no data describing this group of patients. For these patients, waiting for medical attention can be quite dangerous. Unfortunately, this type of life-threatening mis-triage can be found in every triage system in the world^6^.

Currently, in all major triage systems, triage is mostly performed by experienced nurses based on a mix of subjective (e.g., a patient’s level of distress) and objective (e.g., vital signs) criteria. There will be some pre-established ‘red-flag’ values for these criteria, such as a systolic blood pressure below 90 mmHg, loss of consciousness, or complaints of chest pain, etc. However, there are some patients whose vital signs or medical histories do not cross these thresholds, and further improvements to the identification of critical patients by considering patient age, sex, multiple vital signs, ED arrival mode, etc. may be possible. Artificial intelligence approaches may have advantages in such complex, non-linear situations^7–9^.

This study aimed to derive and then validate a machine learning method to support the identification of potentially life-threatening mis-triage and offer at triage a real-time, detailed explanation showing why the algorithm scored a patient as high risk in the hope of improving the detection of ED patients in need of critical care.

## Methods

We analyzed the ETS database from the ED of a major tertiary-care urban teaching hospital for this study. It is not a trauma center and has approximately145 thousand ED visits per year. An electronic ETS (EETS) classification started being used in 2012^10,11^. The ED triage staff inputs basic information about patients into the EETS to arrive at the “traditional” ETS emergency triage level classifications. EETS uses the four-tier ETS algorithm: immediate (level 1), emergent (level 2), urgent (level 3), and non-urgent (level 4). EETS also collects patient age, sex, arrival mode, arrival time, triage vital signs (pulse rate, systolic and diastolic blood pressure, respiratory rate, temperature, and oxygen saturation), level of consciousness, chief complaint(s), blood sugar level and visual analogue (pain) scale (VAS). EETS data from November 2012 to December 2019 were available for analysis. We excluded patients who were dead on ED arrival, or who left before being triaged. During feature engineering, we used the value of -1 to represent the group of missing data and excluded those data categories with > 50% missing information or clearly spurious data (systolic blood pressure > 300 mmHg, diastolic blood pressure > 200 mmHg, pulse rate > 300/min, or oxygen saturation > 100%). The ED resuscitation room was the site of monitoring for patients who needed critical care or immediate treatment. Final diagnosis data was collected using the International Classification of Diseases, Tenth Version (ICD-10) codes for each patient. What major treatments were performed in the resuscitation room were also noted, which was obtained from local Resuscitation Room Database, including ventilator use, vasoactive drug use, or major (invasive) procedures. Resuscitation room mortality was also recorded.

We used CatBoost Python package (open-source, Russia) as a training model for the prediction of mis-triage^12,13^. We implemented a pre-science-like approach^10^ in predicting whether patients were going to be a “life-threatening mis-triage” case. This approach can give a patient’s overall relative risk of such mis-triage as well as the relative risk features of the case leading to the MLS protocol’s positive call to aid ED staff (see Fig. S1).

Based on 8 years of retrospective data, our machine learning system was designed to identify “life-threatening mis-triaged patients”, defined as those patients who were initially triaged as level 3 or 4 by EETS, but were then admitted to the resuscitation room within 24 h. In the development phase of our proposed MLS, we used all positive ‘life-threatening mis-triaged’ cases from the dataset, while negative cases were randomly selected from the same dataset. After training and validation, the MLS first outputs a relative risk (Odds ratio in log(e) space) of whether a patient should be admitted in resuscitation room immediately. In addition, the MLS also outputs the relative risk of every single feature collected at triage, so the medical staff can know why the patient should be admitted to the resuscitation room.

To be specific, we used SHAP^14^ (SHapley Additive exPlanations), a game theoretic approach to explain the output of an MLS model based on the training datasets. We used the SHAP approach to delineate the effect of important features for groups and individuals to the overall MLS algorithm. The SHAP results are shown Fig. 3 and the Pearson correlation of numerical features and labels is shown in Fig. S2.

**Figure 1:**
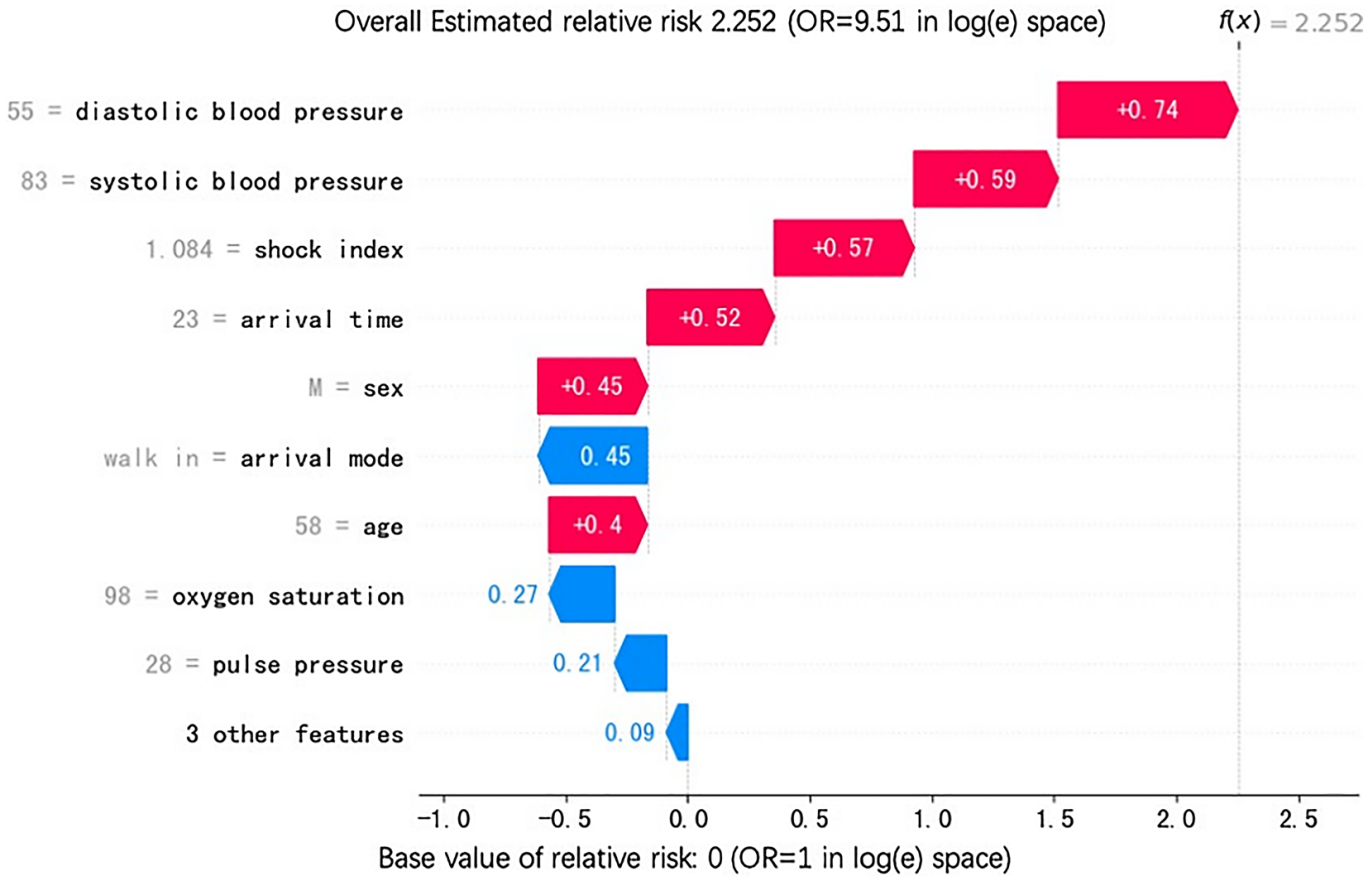
An example of an individual’s relative risk results.

In the prospective cohort design, we randomly selected 30 days between March 1 and April 29, 2020 as a control group using the standard EETS protocol, while in the remaining 30 days between March 1 and April 29, 2020, our MLS protocol was adopted as an intervention group. During the prospective cohort phase, in the intervention group, for better use of the MLS, we provided an individual’s odds ratios (ORs) to help further triage (an example is shown in Fig. 1). Once the patients were flagged by the MLS in the intervention group, triage nurse would call a supervising ED physician for aid, whether they will be in the resuscitation room depends on the decision of the supervising ED physicians. The study was approved by the hospital’s ethics review committee prior to the study commencing.

### Ethics approval

The study was approved by the Ethics Committee of Peking Union Medical College Hospital with a waiver of informed consent from the patient.

### Statement

We confirm that all methods were carried out in accordance with relevant guidelines and regulations on this website: The EQUATOR Network|Enhancing the QUAlity and Transparency Of Health Research (equator-network.org).

## Results

Between November 1, 2012 to December 31, 2019, there were 1,023,613 ED visits, for which the EETS triage-level information is shown in Table 1. The ‘life-threatening mis-triage’ rate in this group was 1.12 ± 0.04%. Most of the ‘life-threatening mis-triage’ cases (5624 out of 7382, 76.19%) admitted to the resuscitation room within 4 h after triage. The top 20 final diagnoses of patients entering the ED resuscitation room are shown in Table 2. The rate of ventilator use for patients in the resuscitation room was 29.6%, vasoactive drug use was 30.2%, invasive procedure (endoscopic therapy、interventional therapy、surgical treatment, et al.) rate was 32.5%, and resuscitation room mortality was 8.6%.

**Table 1:** Patient characteristics.

Characteristics	Development and validation set	Prospective test set
Years–months	2012.10–2019.12	2020.03–2020.04
Arrival time distribution		
Total no. of cases	22,272	17,072
Age, years: median (25th, 75th percentiles)	53.0 (34.0, 67.0)	51.0 (34.0, 65.0)
Female (%)	55.3%	53.9%
ETS level 3 (%)	48.9%	47.7%
Systolic blood pressure	122.0 (109.0, 138.0)	125.0 (113.0, 140.0)
Diastolic blood pressure	75.0 (66.0, 84.0)	77.0 (68.0, 86.0)
Heart rate	88.0 (76.0, 102.0)	89.0 (78.0, 102.0)
Oxygen saturation	99.0 (97.0, 100.0)	99.0 (97.0, 100.0)
Shock index	0.7 (0.6, 0.9)	0.7 (0.6, 0.8)
Pulse pressure	46.0 (36.0, 60.0)	47.0 (38.0, 60.0)
Altered mental status	102 (0.4%)	21 (0.1%)
**Arrival mode**		
Ambulance	3555 (16.0%)	647 (3.8%)
Non-ambulance	18,717 (84.0%)	15,518 (96.2%)

**Table 2:** The top twenty final diagnoses for patients entering the resuscitation room.

Diagnostic category	Proportion (%)
Acute coronary syndrome	9.4
Acute cerebrovascular disease	7.8
Abdominal pain	6.9
Pneumonia	6.5
Gastrointestinal hemorrhage	5.3
Other lower respiratory diseases	5.2
Shock	4.7
Congestive heart failure	3.9
Cardiac dysrhythmias	2.8
Syncope	2.7
Trauma	2.5
Diabetes mellitus with complications	2.3
Respiratory failure	2.1
Sudden death	1.9
Septicemia	1.8
Fluid and electrolyte disorders	1.6
Chronic obstructive pulmonary disease	1.6
Fever of unknown origin	1.5
Non-specific chest pain	1.4
Poisoning	1.3

**Figure 2:**
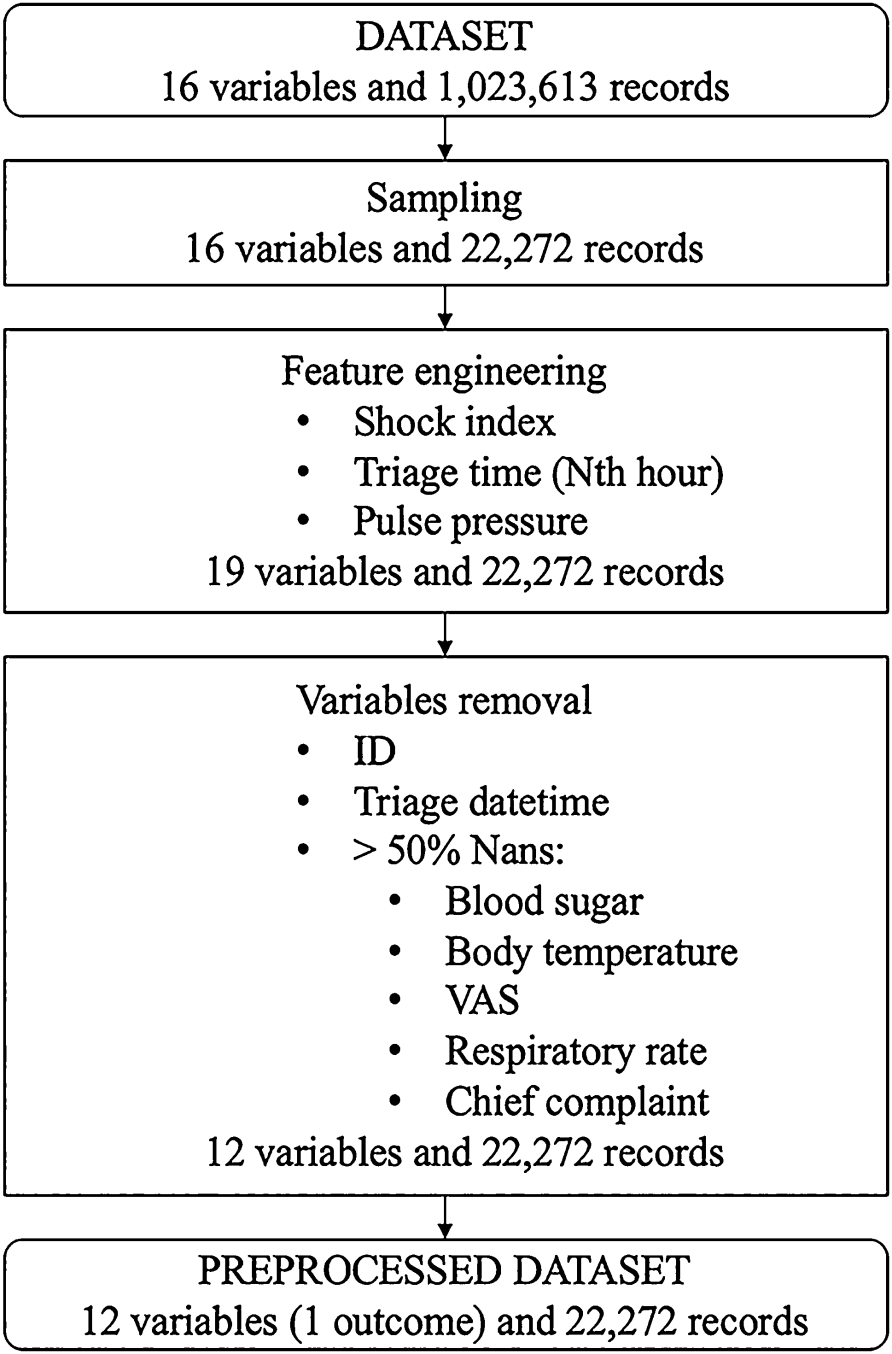
Data processing flow chart.

After data pre-processing (see Fig. 2), 22,272 cases were included for MLS training and validation, including 7382 positive cases and 14,890 negative cases. In the prospective testing phase (March 1 to April 31, 2020) 17,072 cases were included. Statistical analysis was performed to compare difference among different groups of data, where t tests and chi-square test were used for continuous and discrete variables respectively (see Table 3).

**Table 3:** Patient characteristics in different groups for prospective test set.

Characteristics	Control group	Case group	*p* value
Total no. of cases	8839	7936	
Age, years: median (25th, 75th percentiles)	51.0 (34.0, 65.0)	50.0 (34.0, 65.0)	0.18
Female (%)	54.6%	53.1%	0.06
ETS level 3 (%)	47.9%	47.6%	0.45
Systolic blood pressure	125.0 (113.0, 138.0)	125.0 (113.0, 140.0)	0.04
Diastolic blood pressure	77.0 (68.0, 86.0)	77.0 (68.0, 86.0)	0.004
Heart rate	89.0 (78.0, 101.0)	89.0 (78.0, 102.0)	0.57
Oxygen saturation	98.0 (97.0, 100.0)	99.0 (97.0, 100.0)	0.06
Shock index	0.7 (0.6, 0.8)	0.7 (0.6, 0.8)	0.30
Pulse pressure	47.0 (38.0, 60.0)	47.0 (38.0, 60.0)	0.95
Altered mental status	12 (0.1%)	9 (0.1%)	0.7
**Arrival mode**			0.4
Ambulance	321 (3%)	314 (4%)	
Non-ambulance	8518 (97.0%)	7602 (96.2%)	

Considering the degree of missing, only 16,755 out of 17,072 cases are included for use during the prospective testing phase.

### MLS performance

Some of the features included for mis-triage prediction are categorical features, which is not necessary comparable with each other, thus unable to be used in binary decision trees directly. Catboost, powerful implementations for gradient boosting, targeting at handling categorical features, is suitable for establishment of our mis-triage MLS.

Using fivefold cross validation, the MLS protocol achieved an AUC of 0.875 ± 0.006 (CI:95%) on the retrospective dataset (see Fig. S3). Compared with logistic regression (LR) model (AUC: 0.843 ± 0.005 (CI:95%)), our model achieve a satisfactory performance with more than 3% increase (see Figs. S3 and S4). The calibration plot of our MLS and reference model shows that there is no significant prediction tendency for both (see Fig. S5).

**Figure 3:**
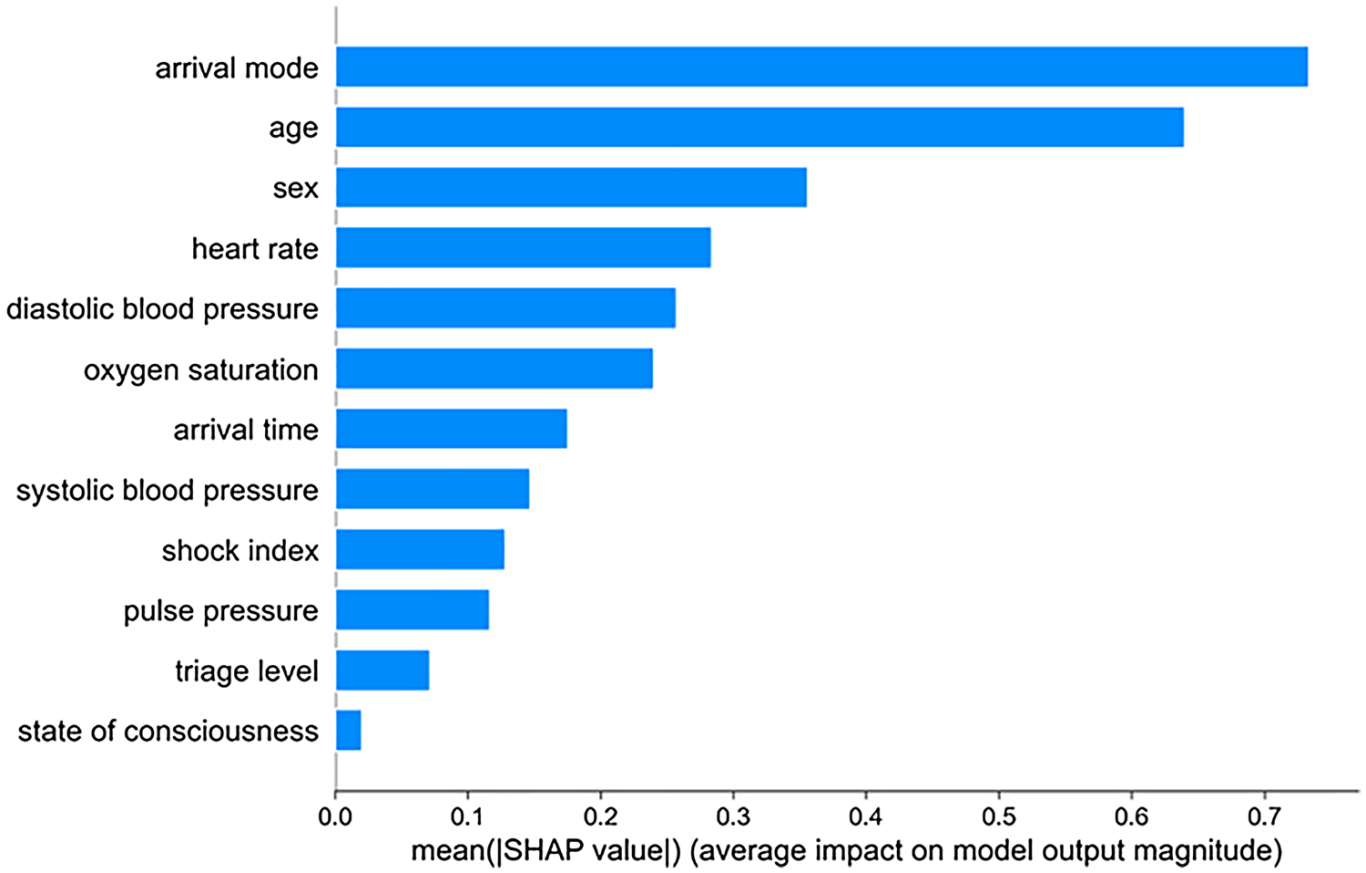
The impact of each model element to the overall model.

The baseline ‘life-threatening mis-triage’ rate in the prospective control group was 1.2% (110 out of 8839) and 0.9% (72 out of 7936) in the intervention group, showing statistical significance with the chi-square test result (*p* = 0.037), while the number of times a triage nurse called a supervising ED physician for aid in a triage decision increased 24.3% (1836/30 day vs 2283/30 day) in the intervention group compared to the control group (Fig. 3).

This MLS model integrates arrival mode, age, sex, and arrival time into the system, none of which are included in the current emergency triage standard. According to our results, arrival mode, age, and sex were the top-three most important features for the MLS protocol.

We also found that higher or lower heart rates, extreme diastolic or systolic pressures, lower oxygen saturation, older age, and arrival at night (Figs. S6, S7, S8, S9, S10, and S11) were associated with a higher mis-triage risk. In determining whether a triage Level 3 or 4 patient needs critical care, the shock index and pulse pressure were important triage features both to the MLS and to traditional triage (see Figs. S12 and S13). In further analysis, shock index has a higher sensitivity in older patients while being less sensitive in younger patients (see Fig. S14). On the other hand, pulse pressure has a higher sensitivity in younger patients while being less sensitive in older patients (see Fig. S15).

## Discussion

Based on 1,023,613 visits, we built an MLS protocol with a satisfactory AUC to test for the odds of a patient with an initial ETS Level 3 or 4 encountering a ‘life-threatening mis-triage’ situation and requiring up-triage into the resuscitation room during their ED stay. At the same time, we used interpretability as an essential practical reference-point for triage officers, so that medical staff may intuitively understand the weight of each triage feature in the conclusions given by the MLS. By being more ‘understandable’ than a simple ‘black box’ system, we hoped this MLS would better aid in making triage decisions. Used in a prospective dataset, we found a significant decrease in the ‘life-threatening mis-triage’ rate, demonstrating its effectiveness and usefulness.

Current clinical triage systems, including the ESI in the United States and the ETS in China, have significant limitations. They rely to some degree on the subjective judgment of triage staff, which has led to significant variability in triage results^15^, especially in pediatric patients^16^. About half of the ED patients in the United States are triaged as ESI Level 3^17^, and many of these patients have conditions that potentially are quite emergent^18^. The mortality rate of ESI level 3 patients in most triage systems is not low, even reaching nearly half of that of level 2 patients^6^. In China, most patients are triaged into ETS Level 3 or 4^5^. In our retrospective study, the rate of ‘life-threatening mis-triage’ in ED patients with initial ETS Level 3 or 4 was 1.12%. Reducing this rate may save lives. For a typical ED with 100,000 patient visits a year, reducing the mis-triage rate to 0.9% with an MLS as we were able to do in this study, would translate into hundreds of fewer mis-triage cases.

Machine learning can modify its response patterns by creating data systems, developing algorithms, and applying them to inference outcomes from new data, which is most applicable to a number of complex, nonlinear relationships. Machine learning models have been applied to predict outcomes in different medical fields, such as predicting hypoxemia during surgery^10^, predicting mortality in patients with sepsis^19^ acute cardiac complications in patients with acute chest pain^20^, and the likelihood of severity and hospitalization in children^21^ and adults^22^ with asthma or COPD exacerbations, respectively. Previous studies have also looked at adult ED triage to predict patients' risk of critical illness or hospitalization^8,23^, but these studies did not provide any explanatory interpretation on the clinical side, making it difficult for MLS results to be accepted by medical staff and integrated into clinical practice. Although relatively straightforward, providing the MLS reasoning allowed us to prospectively validate this MLS protocol and directly test its utility on ‘real-life’ triage cases. Compared to previous studies, our approach provides a detailed indication of a patient’s risk of requiring critical care. In our study’s MLS protocol, each patient is given an individualized odds ratio with the specific weight of each triage feature used in artificial intelligence decision making listed and displayed. Such a descriptive read-out provides a triage nurse with additional material to confidently call a supervising ED physician to assess a patient's condition. In our prospective results, it increased physician consults to tirage by almost 25% and reduced the ‘life-threatening mis-triage’ rate to less than one percent. Traditionally, because of the high workload of ED nurses and physicians, unless a patient's condition differed significantly from the electronic grading system, triage nurses were reluctant to call a physician for consultation. Our MLS can give triage nurses more confidence to make such a call.

Machine learning has some unique advantages in triage. Factors can now be included that were traditionally left out of formal triage protocols. For example, a patient’s time of arrival are important^24^, and a patient’s mode of arrival is also anecdotally acknowledged as being important by experienced triage nurses: patients that arrive on ambulances or arrive at night tend to be sicker. In fact, in our MLS results, the most important triage feature was arrival mode, which is similar to other machine learning triage studies^8^. Age is also an essential parameter in most critical care scoring systems, as older patients often have more underlying diseases and poorer prognoses than younger patients^25^. However, age cutoffs in traditional triage systems tend to be too blunt. For example, the risk profile of a 64-year-old compared to a 65-year-old is quite similar, despite a cutoff at 65^26^. An MLS protocol that uses age as a continuous variable rather than simple cutoffs may provide better predictive power for ‘edge’ cases. Similarly, in the traditional triage system, it is often exhausting to determine the normal value and critical value of heart rate for pediatric patients of different ages. On the other hand, machine learning triage systems can computationally combine a patient's age with heart rate and other indicators to quickly give an appropriate weighting. Likewise, sex is not used as a high-risk factor in traditional triage, but there are differences between males and females in the risk-profiles of many diseases, and a machine learning method can consider a patient's sex in formulating a risk assessment. In our study, sex was one of the top three most important triage characteristics.

We included two additional indicators in our study: pulse pressure and shock index, which are two circulation indicators that have been found to be highly correlated with acute illness severity in previous studies^27–30^ but have not been widely used in emergency triage to date. Our results found these two indicators to be beneficial as emergency triage characteristics, even more important than systolic blood pressure. Interestingly, we found pulse pressure has a lower sensitivity in older patients, which has not been reported before, perhaps because older patients have decreased vascular compliance, making their pulse pressure higher, thus lowering its sensitivity. The situation with shock index was just the opposite, as it has a higher sensitivity in older patients. This may be due to older patients' relatively poor cardiac compensation ability, requiring a higher heart rate to compensate for any decrease in blood pressure.

We should note that some important traditional triage factors were not included in our MLS system, specifically respiratory rate, which was not included due to many missing data values in the dataset, suggesting poor nurse compliance in obtaining this feature. Respiratory rate measurement has been criticized as requiring time and skills for an accurate measurement^31^. Missing data values for temperature were also large, possibly because most patients had a normal temperature or did not require a routine temperature check, and possibly because more ‘traditional’ temperature measurements are more invasive and time-consuming for triage staff. Chief complaint is clearly important but was also not included because of the large variability in complaints and many missing values. Chief complaints are unfortunately quite subjective, and future MLS acquisition and standardization techniques using very large datasets will likely be needed to better include this as a viable triage factor in the future. All these missing data values suggest that the EETS system may need to change, and some threshold indicators (e.g., shortness of breath, ischemic chest pain, etc.) can possibly be used instead. Interestingly, the non-inclusion of these indicators did not affect the validity and usefulness of this study’s results. On the contrary, the results of this study are more generalizable because we only used easily obtainable, objective indicators.

### Limitations

Our study has a few limitations. Some features we used in the model such as arrival mode, arrival time, and initial emergency triage level can be influenced by local system factors in different geographic areas that use different emergency triage standards. However, MLS can use data from different hospitals to learn quickly and update, thus better meeting the needs of specific hospitals. Secondly, our prospective validation dataset only had 2 months (March and April 2020) of data to evaluate. We plan to continue to examine the MLS results in different seasons in the future. Thirdly, we only focused on patients triaged to Levels 3 and 4 in this study, focused on finding ‘under-triaged’ patients in this group. Future work can examine rates of ‘over-triage’ in Levels 1 and 2. Lastly, although we found that many factors were important for identifying critically ill patients, because of missing data, we were unable to include respiratory rate, temperature, or chief complaint in the system. As noted earlier, this either means better collection methods are needed for these factors or these factors are best not included as either the lack of uptake by triage staff or their subjectivity make their use in an MLS system unreliable.

## Conclusions

It is challenging to accurately differentiate critically ill from stable patients in the ED, which can lead to life-threatening cases of mis-triage. This study’s MLS was able to reduce the life-threatening mis-triage rate from 1.2 to 0.9% in the prospective arm. Compared to previous studies, our MLS approach also provided a detailed explanation of each patient’s risk of mis-triage, and caused nearly 25% more triage calls to supervising ED physicians. Although MLS protocols can lower mis-triage rates, future prospective studies may shed more light on potential reductions in patient morbidity and mortality.

## Supplementary Information

Supplementary Figures.

## Data Availability

The datasets generated and/or analyzed during the present study are available from the corresponding author upon reasonable request.
