# Supplementary material for: Development and validation of a practical machine-learning triage algorithm for the detection of patients in need of critical care in the emergency department
Source: Sci Rep. 2021 Dec 15;11:24044. doi: 10.1038/s41598-021-03104-2 (PMC8674324; doi:10.1038/s41598-021-03104-2)
Supplement: Supplementary file 1 — Supplementary Figures. [file 41598_2021_3104_MOESM1_ESM.doc]

**Title：**

Development and validation of a practical machine-learning triage algorithm for the detection of patients in need of critical care in the emergency department

**Authors names and affiliations:**

Yecheng Liu, MD^1#^

Jiandong Gao, PhD^2, 3#^

Jihai Liu, MD^1^

Joseph Harold Walline, MD^4^

Xiaoying Liu, MM^1^

Ting Zhang, MM^1^

Ji Wu, PhD^2, 3 *^

Huadong Zhu, MD^1*^

Weiguo Zhu, MD^5*^

**Figure Legends**

Figure S1. Overview of the proposed machine learning system.

Figure S2. Pearson correlation of specific model elements.

Figure S3. Model performance using 5-fold cross validation.

Figure S4. LR performance using 5-fold cross validation.

Figure S5. Calibration plot for our MLS and LR model.

Figure S6. Model interpretation of the correlation between heart rate and mis-triage risk.

Figure S7. Model interpretation of the correlation between systolic blood pressure and mis-triage risk.

Figure S8. Model interpretation of the correlation between diastolic blood pressure and mis-triage risk.

Figure S9. Model interpretation of the correlation between oxygen saturation and mis-triage risk.

Figure S10. Model interpretation of the correlation between age and mis-triage risk. Figure S11. Model interpretation of the correlation between Emergency Department arrival time and mis-triage risk.

Figure S12. Model interpretation of the correlation between shock index and mis-triage risk.

Figure S13. Model interpretation of the correlation between pulse pressure and mis-triage risk.

Figure S14. Shock index has a higher sensitivity in older patients.

Figure S15. Pulse pressure has a higher sensitivity in younger patients (especially with higher pulse pressures).


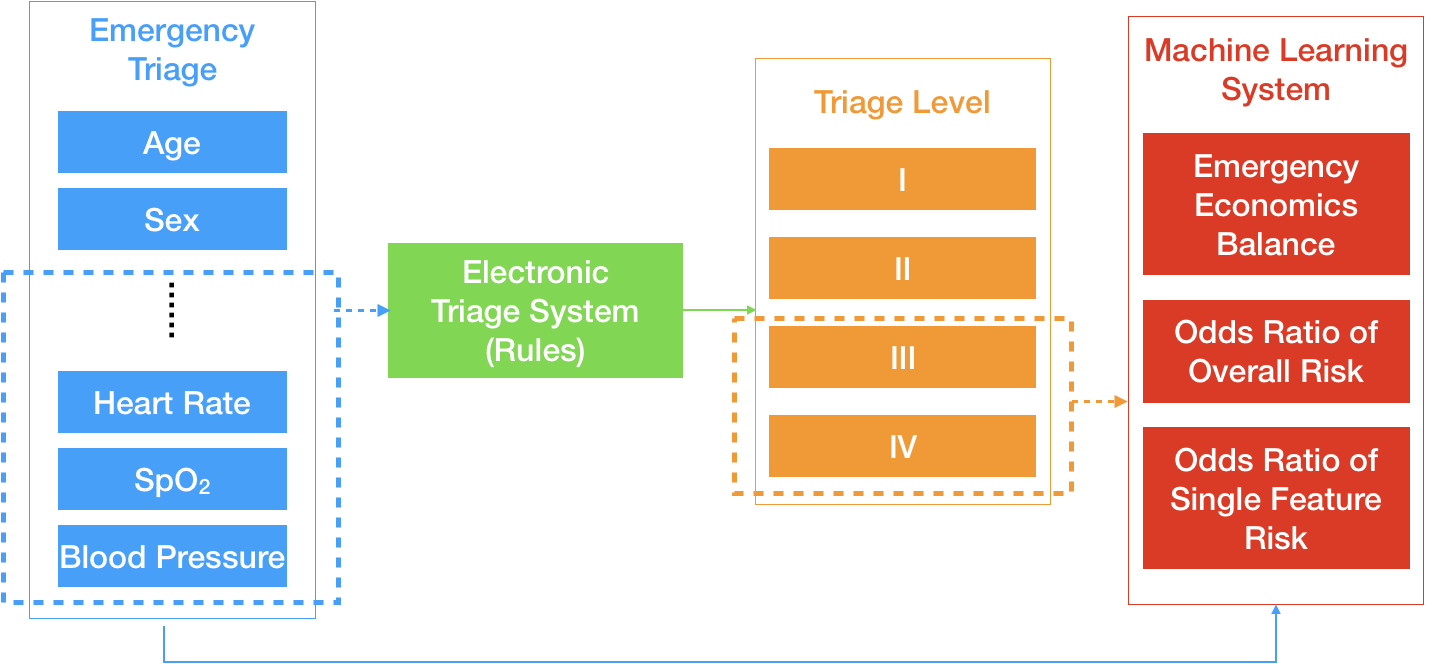


**Figure S1. Overview of the proposed machine learning system.**


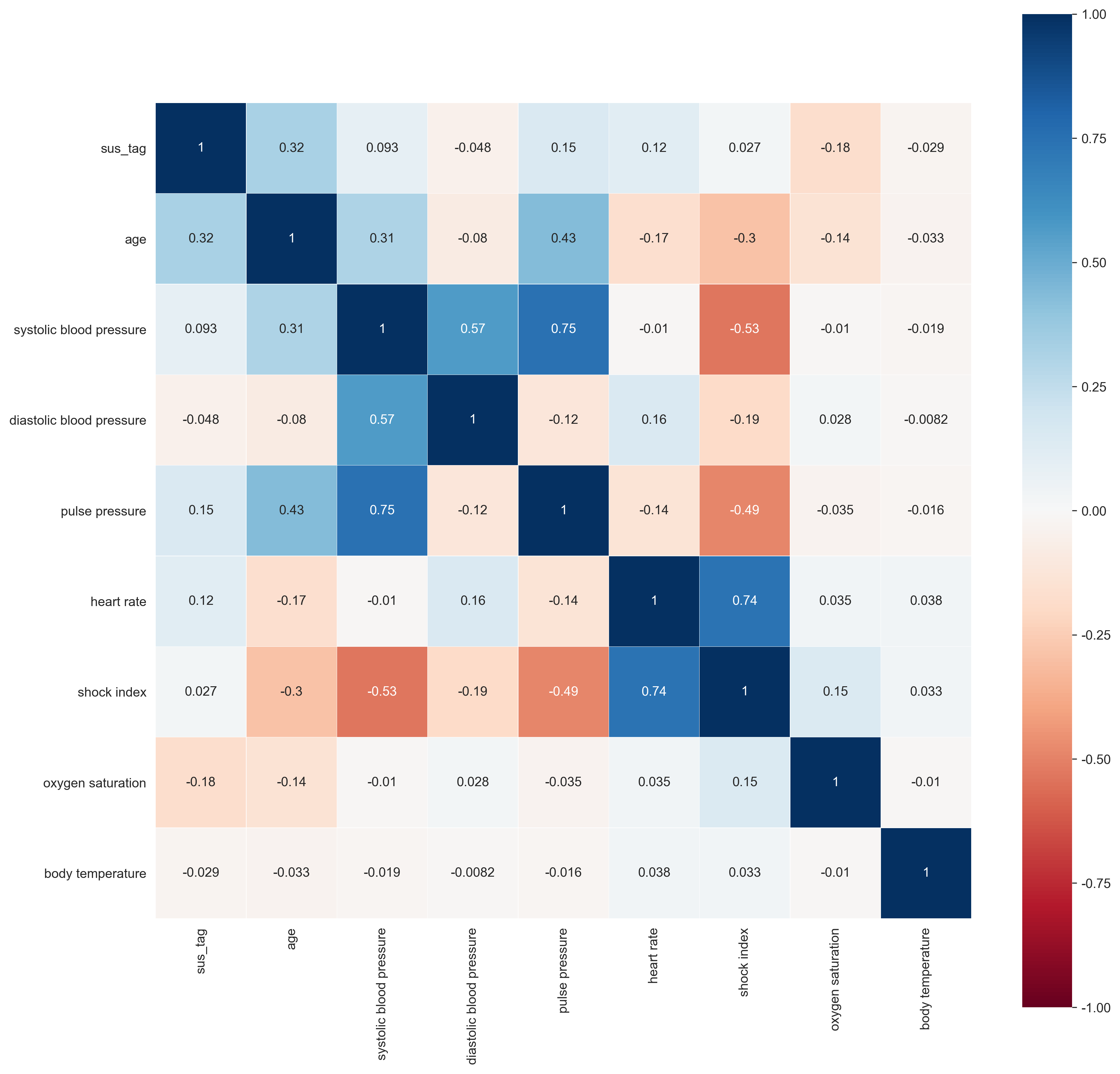


**Figure S2. Pearson correlation of specific model elements**


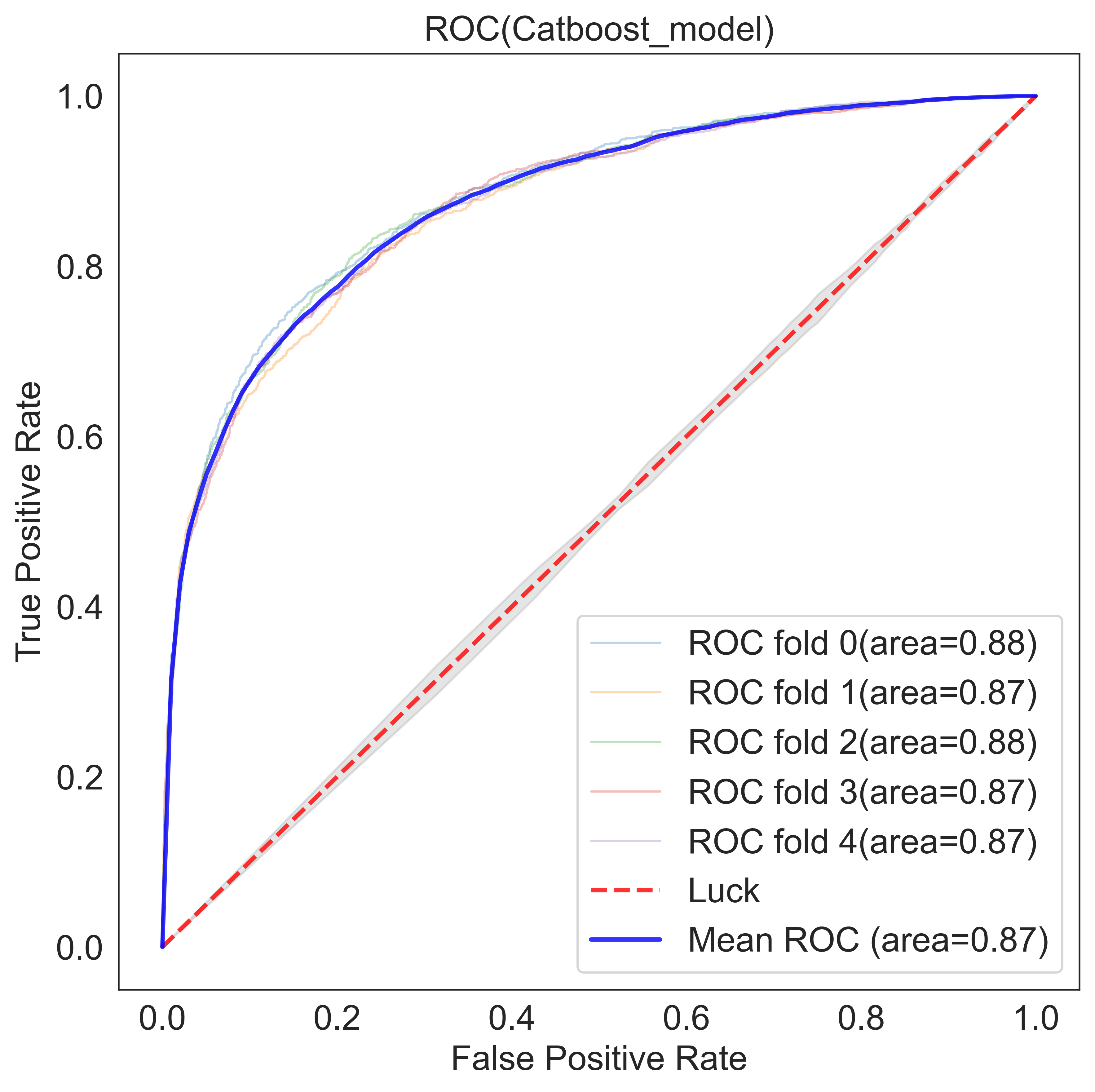


**Figure S3. Model performance using 5-fold cross validation**


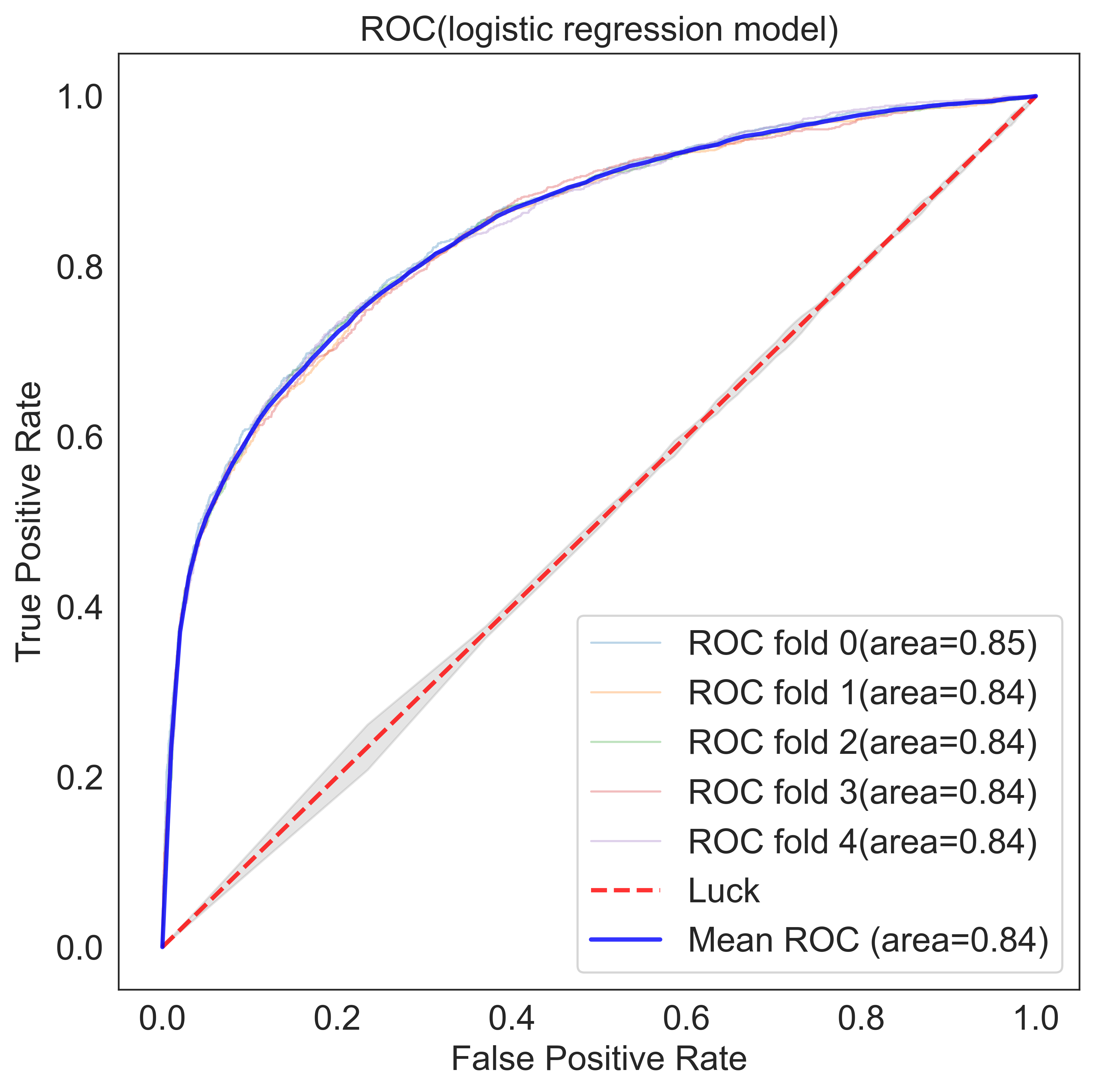


**Figure S4. LR performance using 5-fold cross validation**


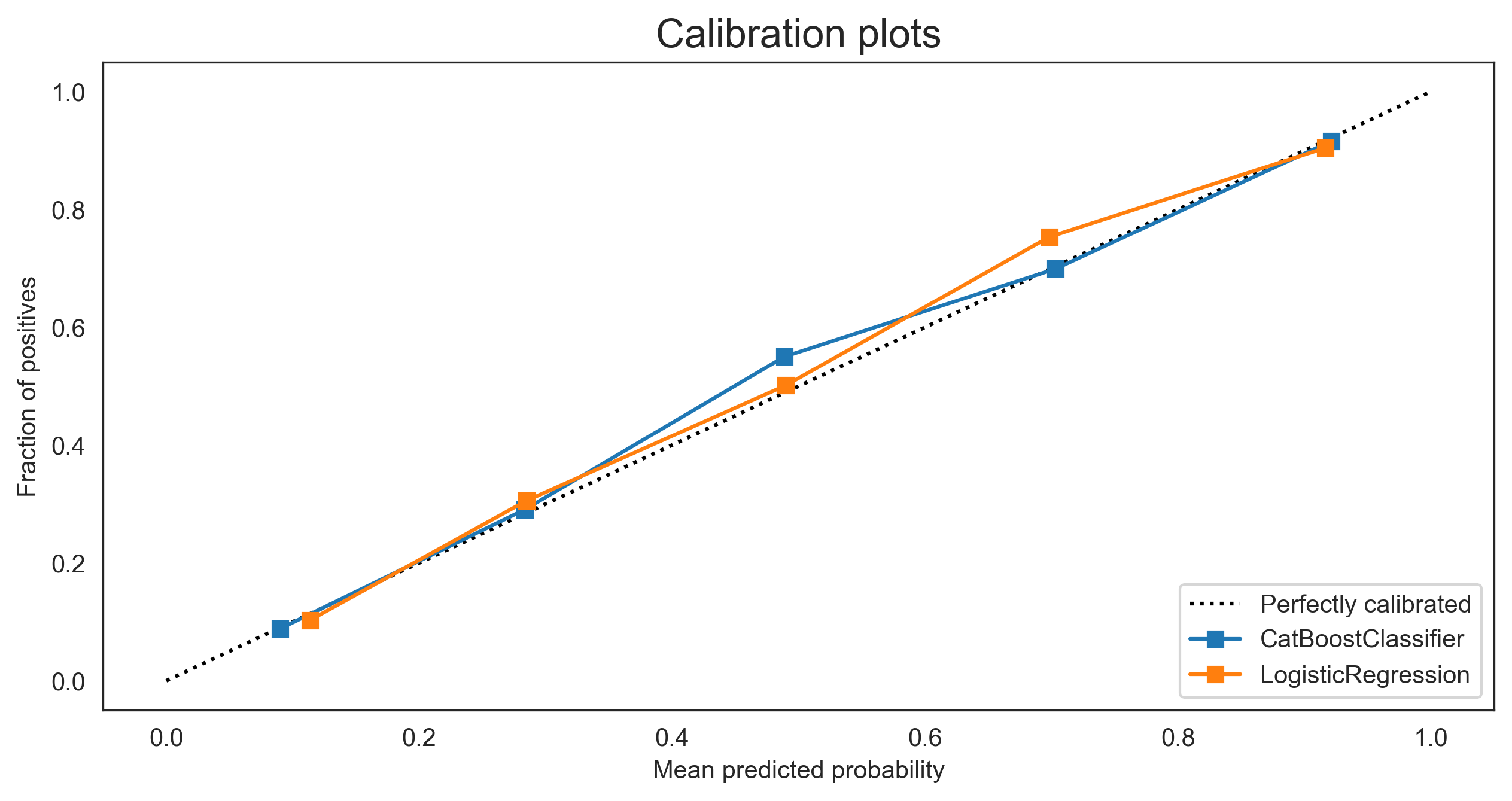


**Figure S5. Calibration plot for our MLS and LR model**


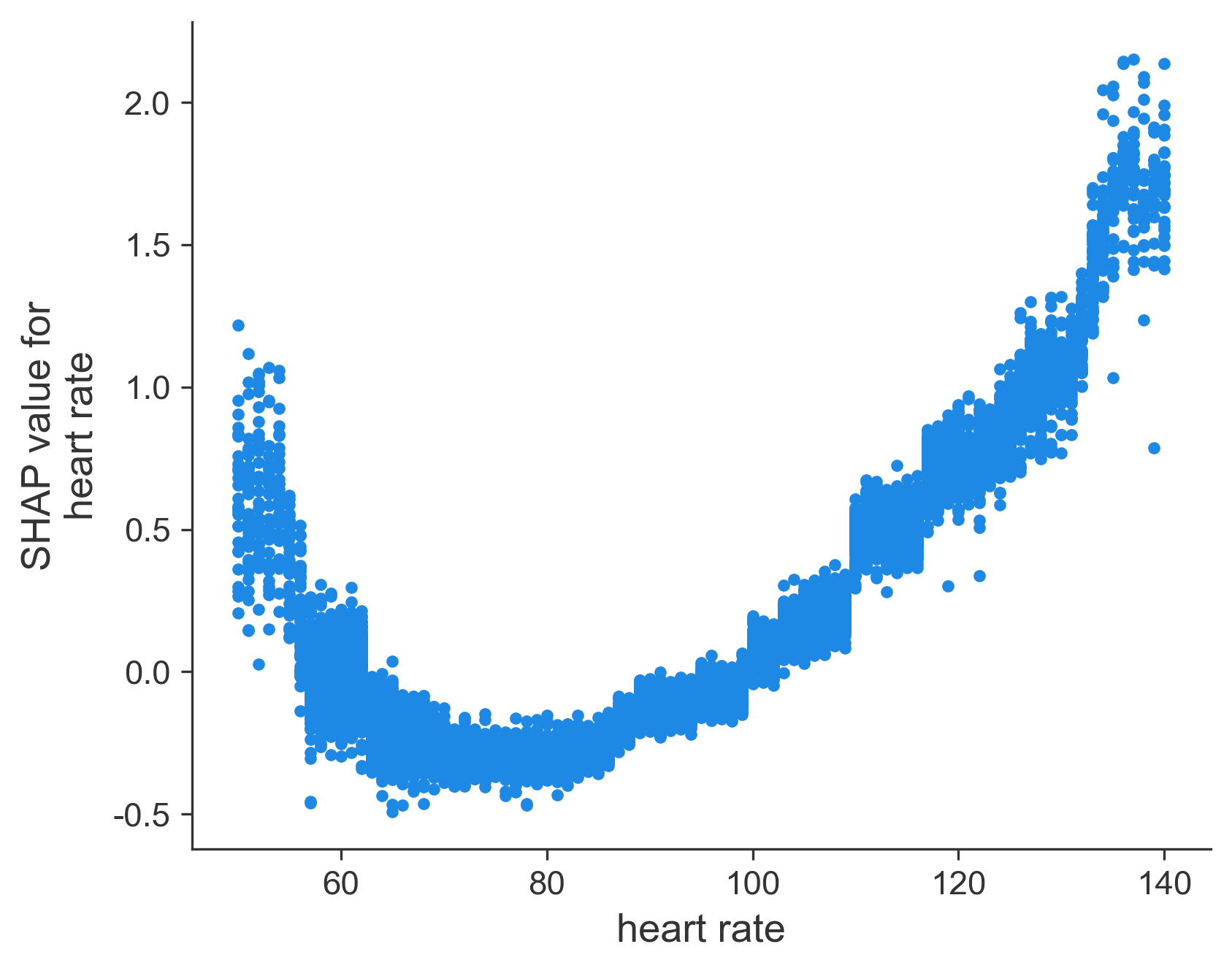


**Figure S6. Model interpretation of the correlation between heart rate and mis-triage risk**


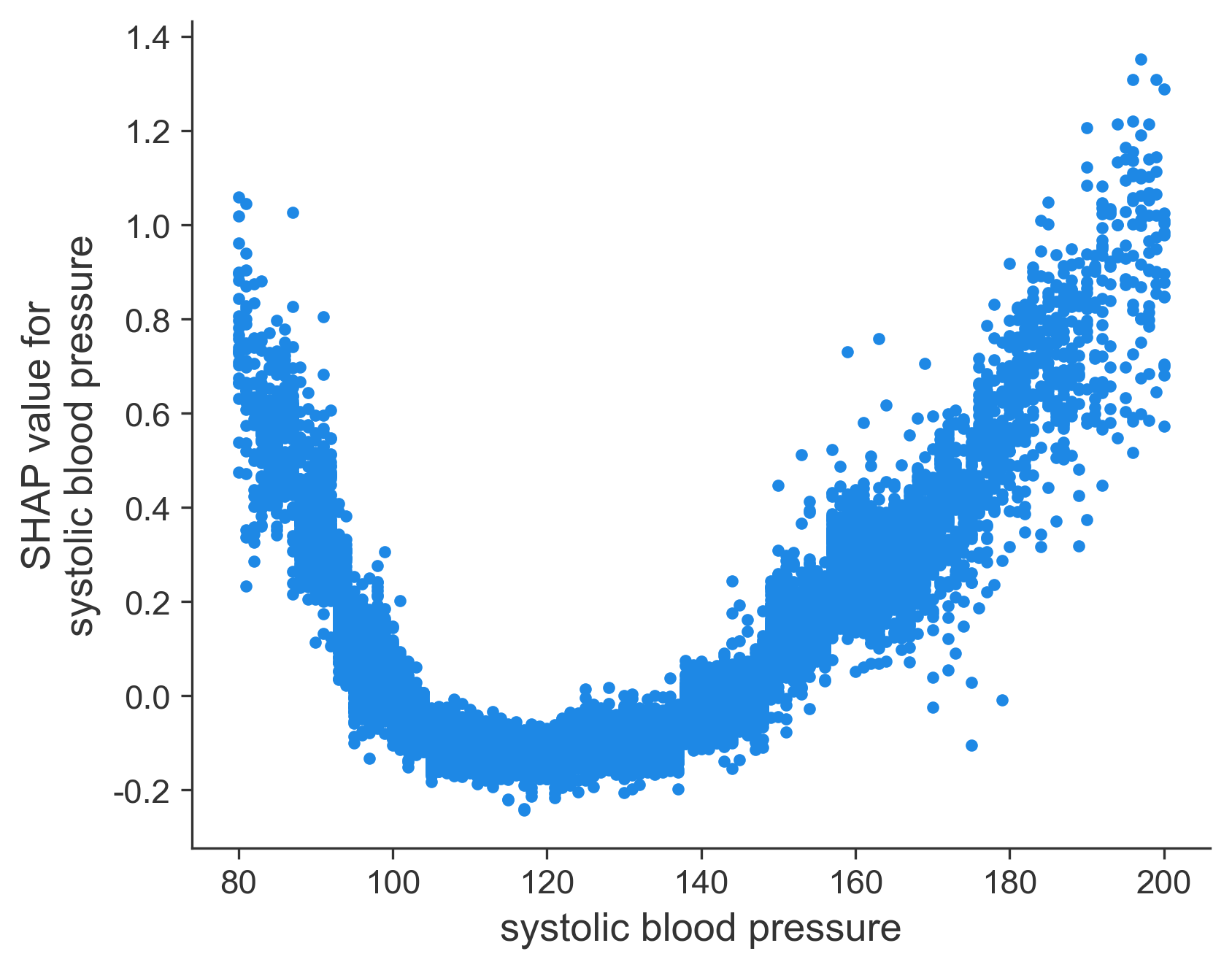


**Figure S7. Model interpretation of the correlation between systolic blood pressure and mis-triage risk**


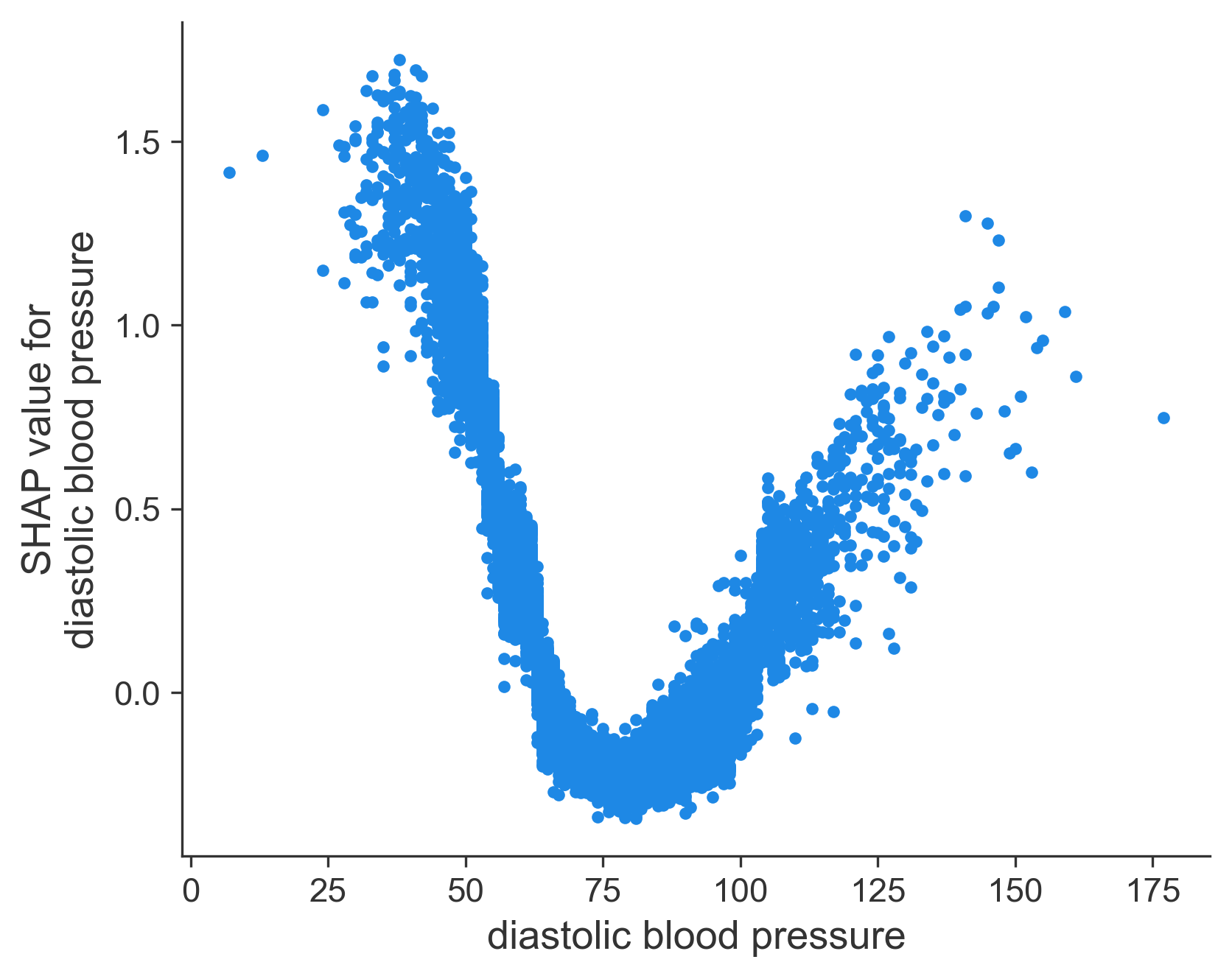


**Figure S8. Model interpretation of the correlation between diastolic blood pressure and mis-triage risk**


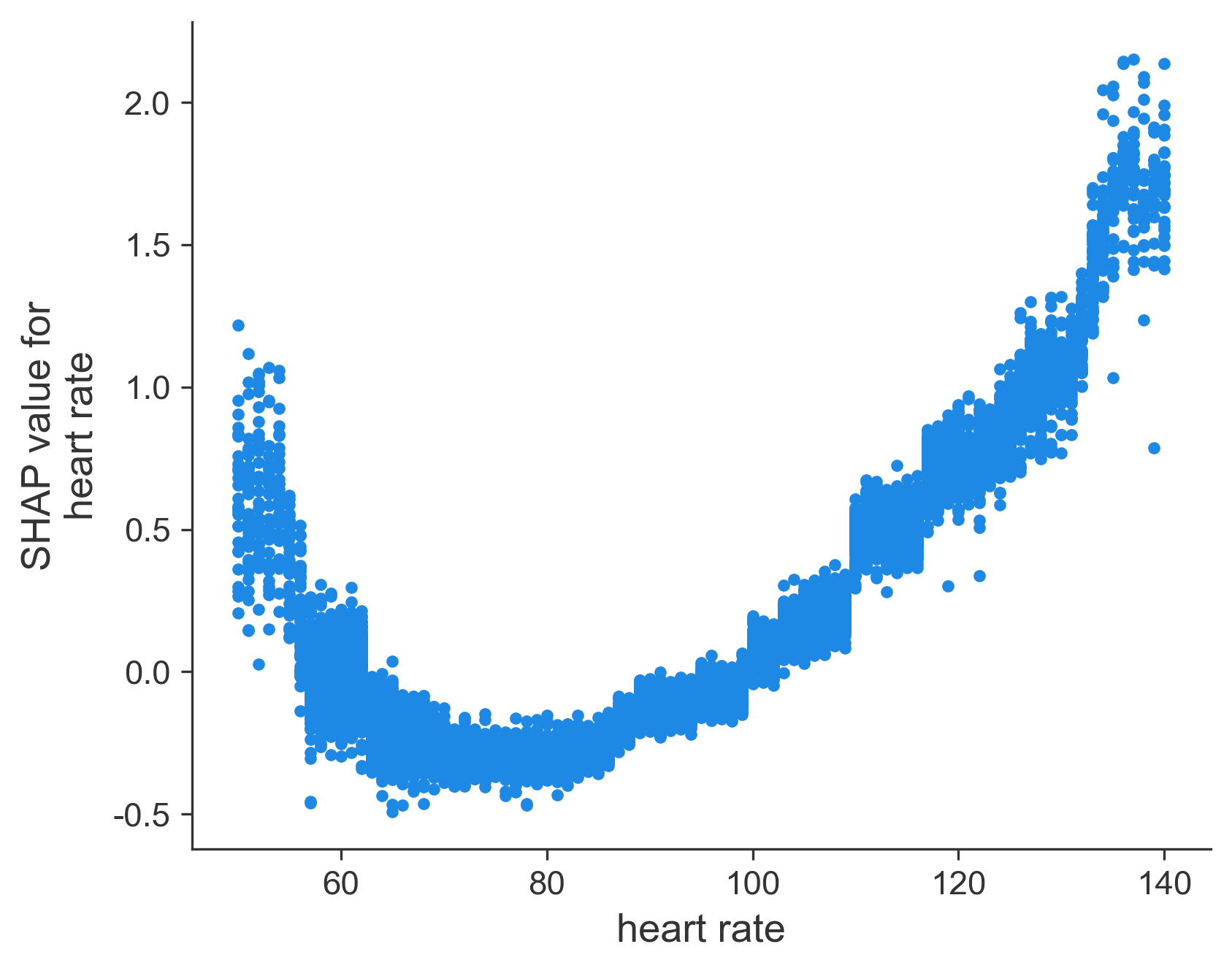


**Figure S9. Model interpretation of the correlation between oxygen saturation and mis-triage risk**


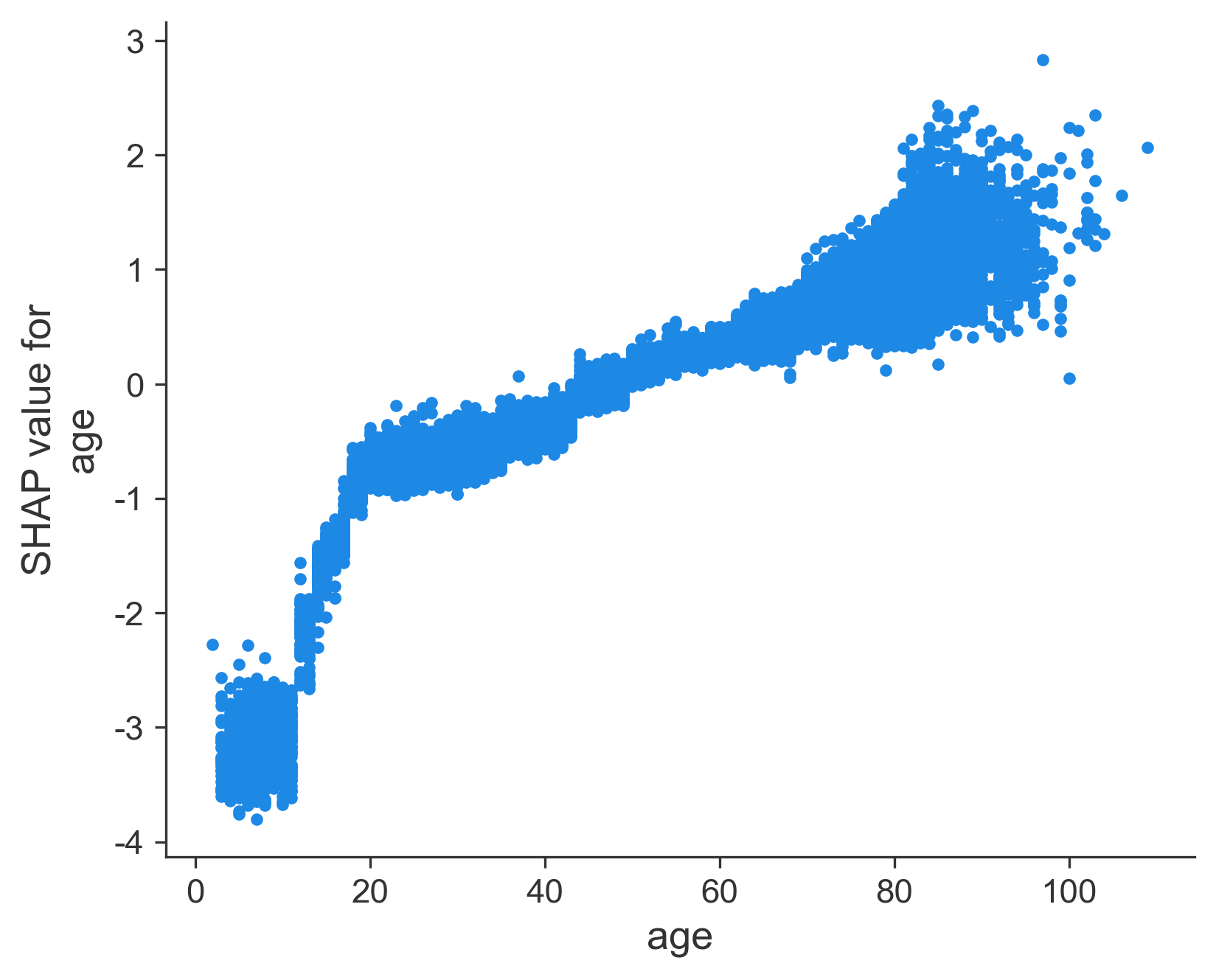


**Figure S10. Model interpretation of the correlation between age and mis-triage risk**


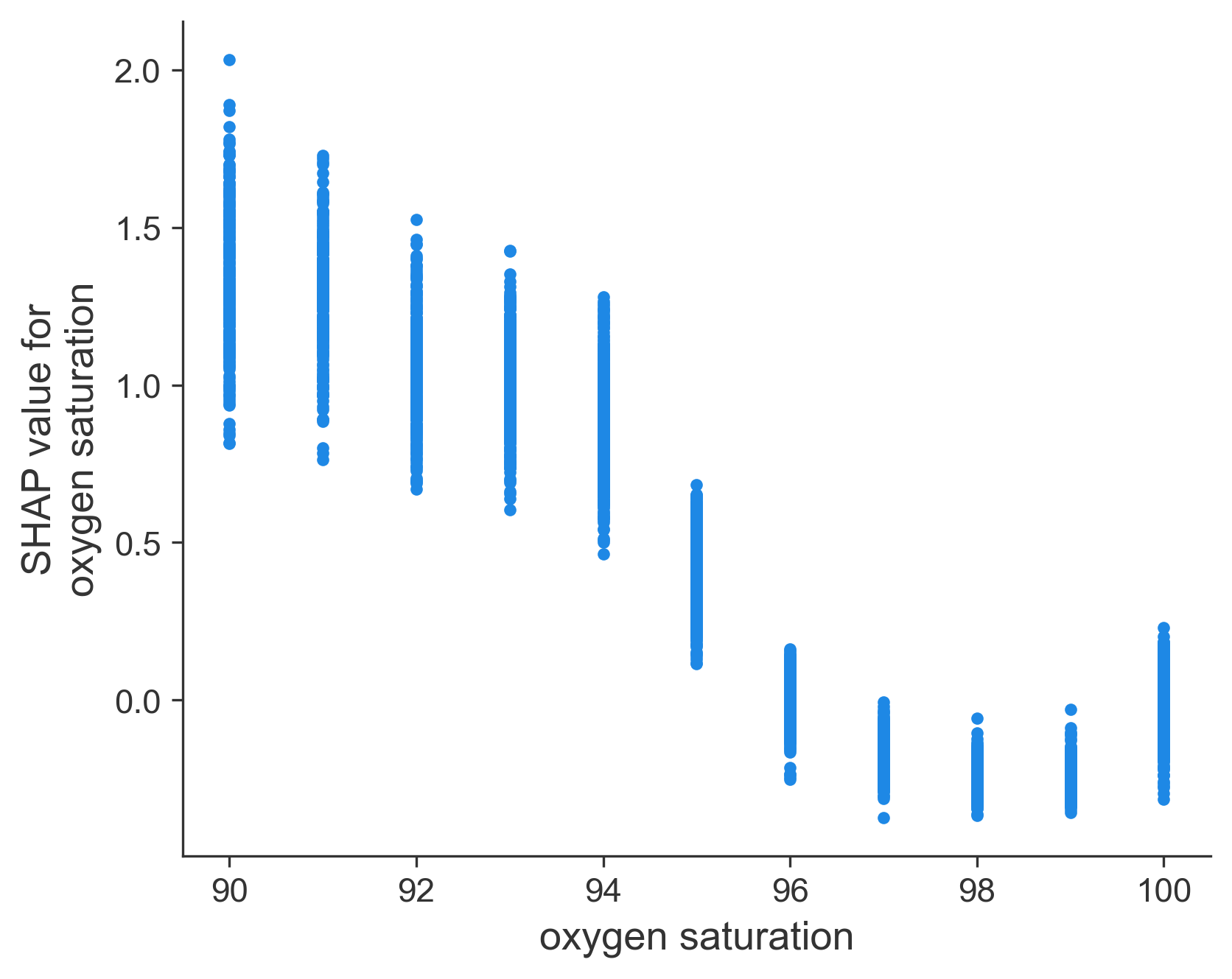


**Figure S11. Model interpretation of the correlation between Emergency Department arrival time and mis-triage risk**


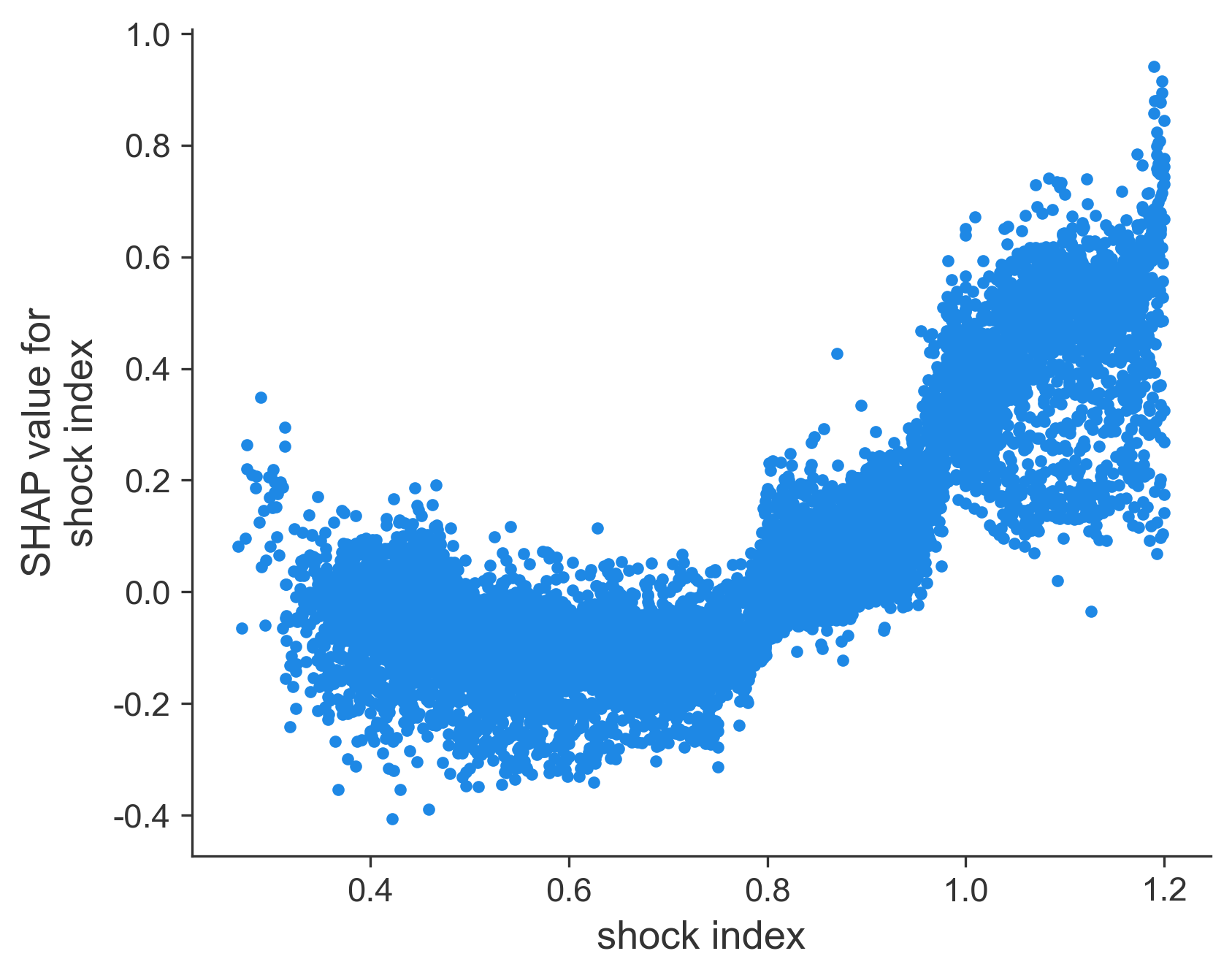


**Figure S12. Model interpretation of the correlation between shock index and mis-triage risk**


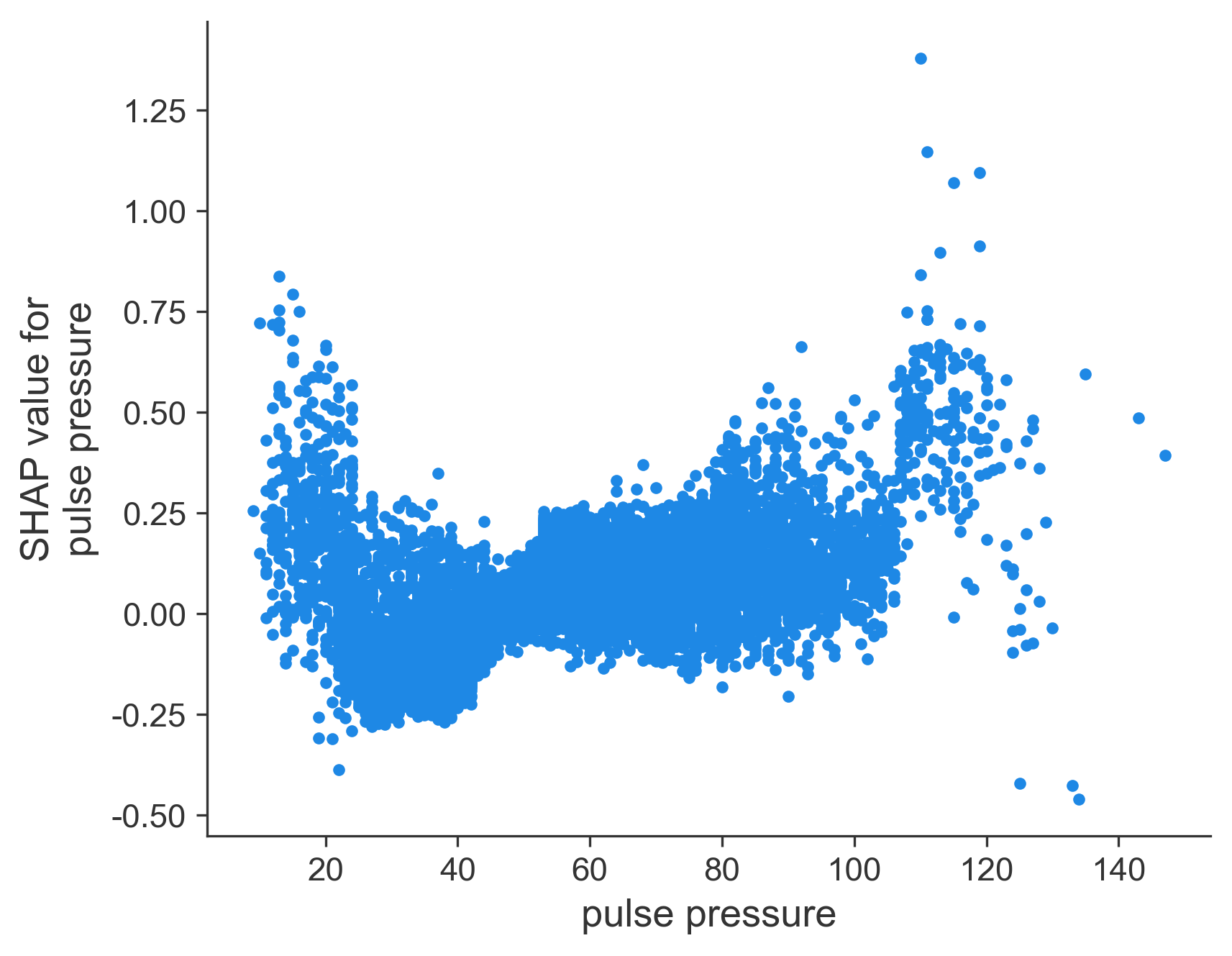


**Figure S13. Model interpretation of the correlation between pulse pressure and mis-triage risk**


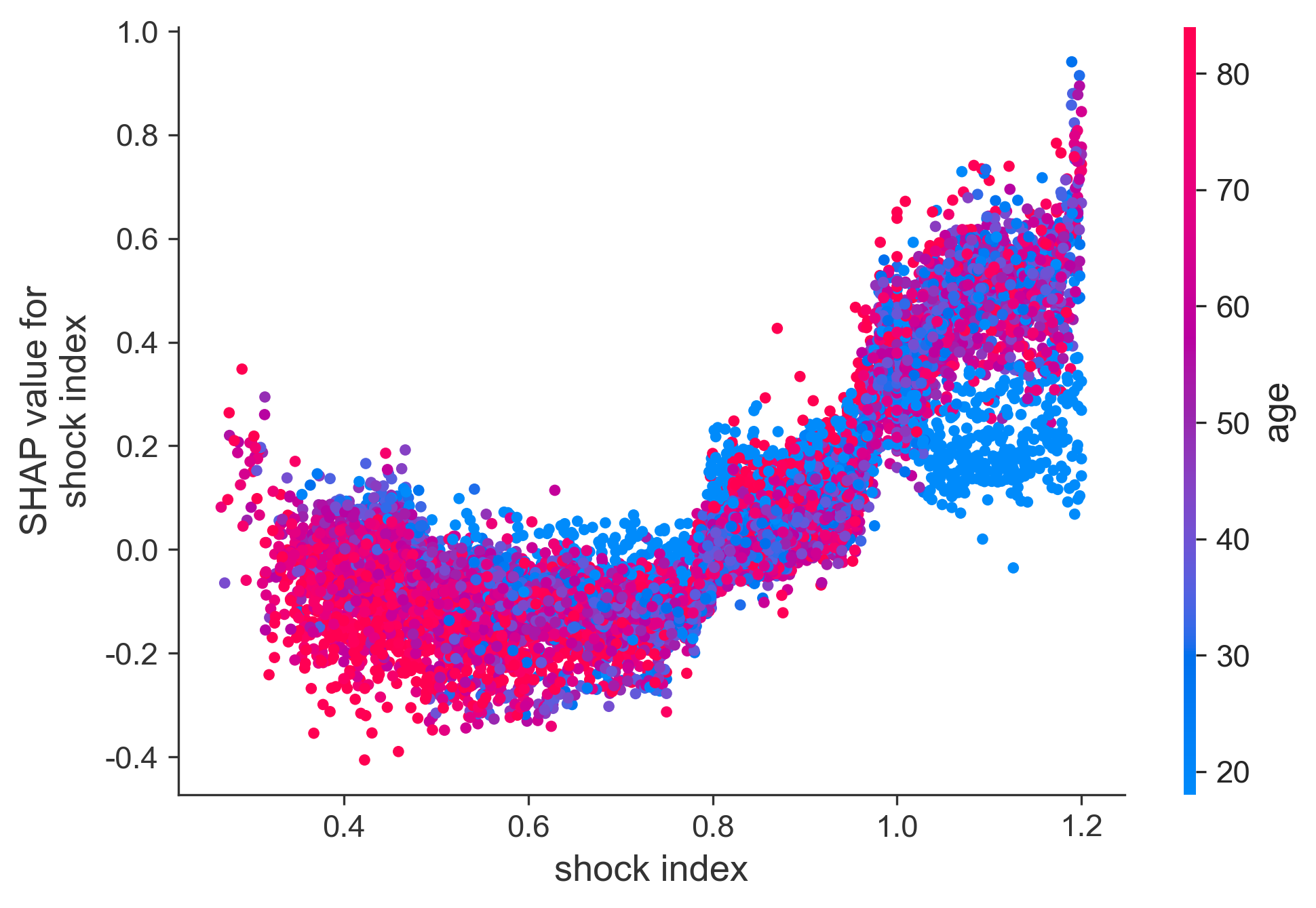


**Figure S14. Shock index has a higher sensitivity in older patients**


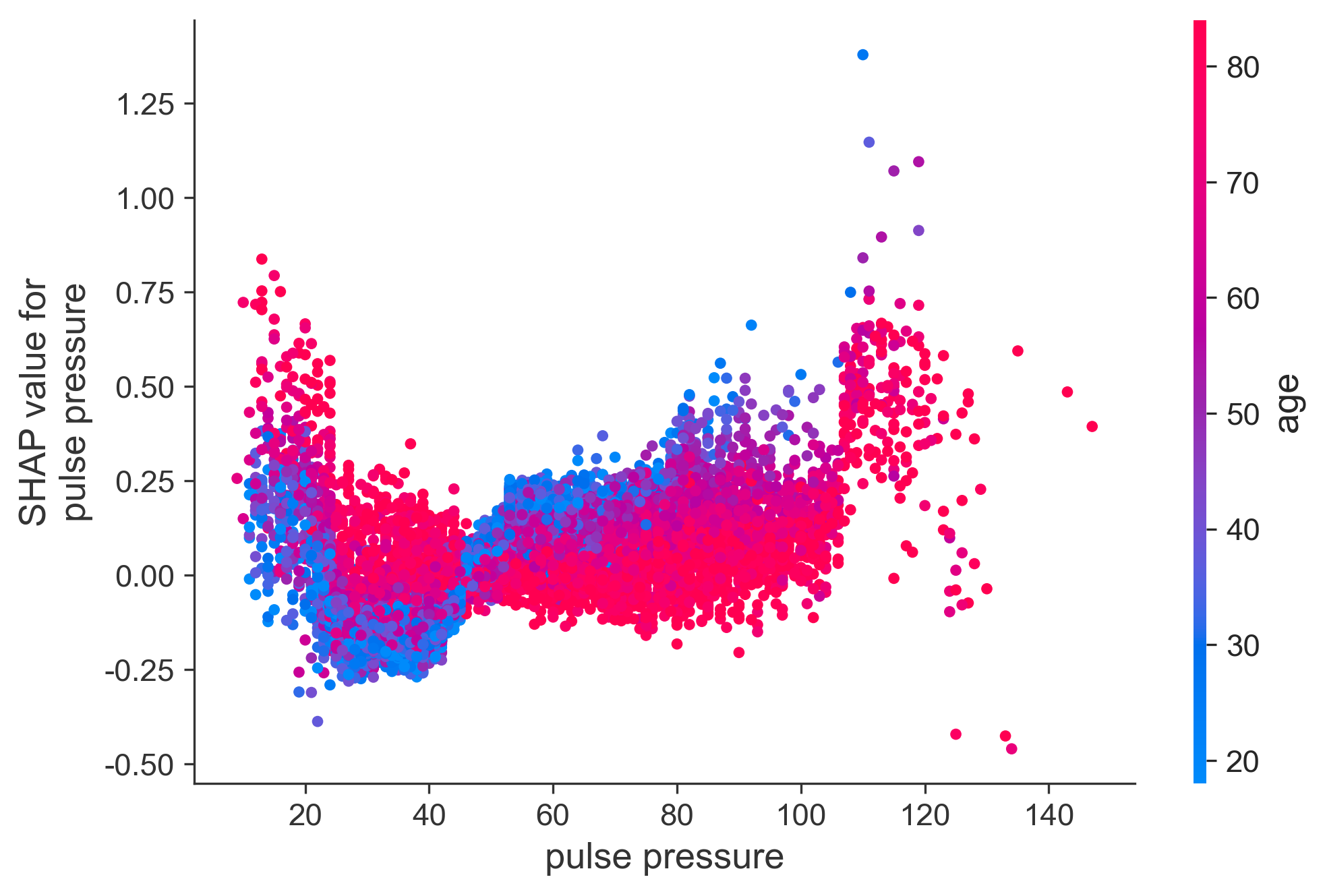
**Figure S15. Pulse pressure has a higher sensitivity in younger patients (especially with higher pulse pressures)**
